# Distance to climate change consequences reduces willingness to engage in low-cost mitigation actions–Results from an experimental online study from Germany

**DOI:** 10.1371/journal.pone.0283190

**Published:** 2023-04-05

**Authors:** Nicolai Heinz, Ann-Kathrin Koessler, Stefanie Engel

**Affiliations:** 1 Environmental Politics, Helmholtz Centre for Environmental Research (UFZ), Leipzig, Germany; 2 Institute of Environmental Systems Research (IUSF), Osnabrück University, Osnabrück, Germany; 3 Institute of Environmental Planning, Leibniz Universität Hannover, Hannover, Germany; 4 School of Business Administration and Economics, Osnabrück University, Osnabrück, Germany; Universidad Central de Chile, CHILE

## Abstract

Adverse consequences of climate change often affect people and places far away from those that have the greatest capacity for mitigation. Several correlational and some experimental studies suggest that the willingness to take mitigation actions may diminish with increasing distance. However, the empirical findings are ambiguous. In order to investigate if and how socio-spatial distance to climate change effects plays a role for the willingness to engage in mitigation actions, we conducted an online experiment with a German population sample (n = 383). We find that the willingness to sign a petition for climate protection was significantly reduced when a person in India with a name of Indian origin was affected by flooding, as compared to a person in Germany with a name of German origin. Distance did not affect donating money to climate protection or approving of mitigation policies. Our results provide evidence for the existence of a negative effect of distance to climate change consequences on the willingness to engage in low-cost mitigation actions. Investigating explanations for such an effect, we find that it can be attributed to the spatial rather than the social dimension of distance. Moreover, we find some cautious evidence that people with strong racist attitudes react differently to the distance manipulations, suggesting a form of environmental racism that could also reduce mitigation action in the case of climate change.

## 1. Introduction

### Climate change is a global phenomenon, characterized by a mismatch of causes and effects

In the context of climate change, those individuals and societies with the highest emissions, and thus also the highest mitigation potential, are often distant to those most severely affected by the adverse consequences of climate change. While most greenhouse gas emissions accumulated in the atmosphere stem from production or consumption activities in the Global North [1], most harm is inflicted on people living in the Global South [2, 3], who can be perceived as distant in terms of physical space (spatial distance) and/or socio-cultural background (social distance). However, distance between causes and effects may diminish the willingness for action. This would pose a particular challenge to mitigate climate change. From a policy point of view, mitigation action could be promoted more effectively by taking into account such a possible effect of distance.

While correlational studies indeed point to an inverse relationship between distance and concern or willingness to act [4, 5], the experimental evidence is more ambiguous [6, 7]. Further research is needed to test a possible impact of distance on the willingness to take mitigation actions and to investigate the mechanisms through which such an effect may take place. Moreover, past research focusses on stated rather than actual behavioral measures, and it does not differentiate impacts of distance on different mitigation actions, which may differ in costliness. Finally, open questions remain with regard to how and for whom distance may affect the willingness to mitigate.

With this paper we aim to address these gaps and add to the understanding of the effect of distance on climate change mitigation actions. First, we analyze the effect of distance to people negatively affected by climate change on several different kinds of mitigation actions. Second, we investigate how such an effect could be explained, in particular whether it is rather due to the social or spatial dimension of distance. Third, we examine for whom such an effect of distance may be strongest. Specifically, we probe for evidence of environmental racism in the sense that people with strong racist attitudes would react more strongly to distance. To these ends, we conducted an online experiment with a German non-student sample (n = 383), where we varied the degree of social and spatial distance to a person adversely affected by climate change induced floods.

Before presenting more details about our study design, we review different theoretical approaches and their shortcomings as they are discussed in the literature to explain an effect of distance. Then, we summarize the empirical findings on the matter so far and discuss different factors that help to make sense of the inconsistent results. Derived from the existing research gaps, we present our research questions and hypotheses at the end of this introduction.

### How could distance impact the willingness to mitigate?–Psychological distance, outgroup derogation and environmental racism

Most studies investigating the effect of distance for climate mitigation actions use Construal Level Theory (CLT) as their theoretical underpinning [e.g. 8, 9]. At its core is the concept of psychological distance, i.e., the proximity relation of an event or person to the self, thus describing the “subjective experience that something is close or far away from the self, here, and now” [10, p. 440]. Both spatial and social distance are different dimensions under the umbrella of psychological distance. CLT posits that greater (vs. lower) psychological distance to an object, e.g. climate change consequences, corresponds to a more abstract (vs. concrete) mental construal of it. People strive to match levels of construal, which will guide their courses of action–e.g. they respond to a distant and thus abstract event with more abstract actions while they respond to concrete events with more concrete actions. It is unclear, however, whether psychological distance as part of CLT is in fact the best explanatory concept for a possible effect of distance on the willingness to mitigate [4, 7, 11]. Brügger et al. [12] have argued that “proximizing” climate change does not directly affect motivation but changes the perceptions and the selection of information that decisions are based on, making the link to action more complex. Thus, the cognitive approach of CLT is not able to clearly predict the effect of distance on mitigation actions.

An alternative explanation is provided by social identity theory [13]. It is based on the idea that individuals categorize others into ingroups (groups they belong to) and outgroups (groups they do not belong to). This categorization often leads to *ingroup favoritism* and *outgroup derogation* [e.g. 14]. While this dynamic even works based on arbitrary or fictional characteristics [15], various dominant social categories exist that structure the world into distinct social groups, e.g. based on nationality, cultural background, religion, gender or other factors. This matters also for the willingness to share own resources to the benefit of others. Two meta studies from experimental economics find that people cooperate less with members of perceived outgroups than members of perceived ingroups [16, 17]. In the context of climate change, it can be expected that the willingness to give up own resources or accept political restrictions for the purpose of climate change mitigation depends on whether the adversely affected parties are perceived as ingroups or outgroups. Distance in terms of space or socio-cultural background could induce such outgroup categorization and derogation. As such, an effect of distance would not be the mere result of a cognitive mechanism, but a fundamental social dynamic that impacts individual and public decisions.

While ingroup-outgroup categorization is based on dis/similarity with respect to a group identifier and regards social groups as existing next to each other, global power structures like White Supremacy (i.e. derogation of racialized people, meaning all people considered as non-white–as compared to white people) positions groups into a hierarchical relation [18]. This could induce another effect of distance on the willingness to mitigate climate change where racialized communities are most severely affected ─ a form of *environmental racism*. So far, environmental racism is mostly discussed with regard to disproportionate exposure to environmental damage by racialized communities [19]. The literature on it, stemming from sociology/geography, has shown with empirically robust results that environmental degradation and exposure to environmental risk disproportionately affect racialized or other marginalized communities [20–22]. Disproportionate exposure to climate change has also been discussed as a racial issue in this context [21]. Such global power structures may not only affect exposure directly, but also indirectly when it comes to individual or collective responses to environmental degradation. As such, environmental racism could be expected to lower the willingness to engage in climate change mitigation efforts in a predominantly white Global North.

For the purpose of our study, we use social identity theory instead of CLT as well as the concept of environmental racism to deduce our hypotheses. While the conceptual approaches of racism and social identity theory are more closely linked to social distance, we also look at spatial distance for two reasons. First, spatial distance may have an effect on its own, e.g. because physical effects of environmental degradation may feel more threatening when they occur in close proximity. Second, acknowledging that both dimensions are intertwined, the use of both distance dimensions enables us to examine which one of them is responsible for a possible effect.

### Spatial and social distance and willingness to engage in mitigation actions–Empirical findings

Existing studies examining whether and how distance impacts the willingness to engage in mitigation actions are of two types: (i) correlational studies (i.e., surveys that elicit both measures of distance and then examine statistical connections to mitigation actions or related measures), or (ii) experiments (i.e., studies that manipulate distance and examine the effect on the willingness to mitigate). While correlational studies on the relationship between the socio-spatial distance to negative climate change impacts and the willingness to engage in climate action consistently point to an inverse relationship between the two, the experimental evidence shows greater inconsistency. Varying socio-spatial distance to climate change consequences seems to have mixed effects on the engagement in mitigation behaviors and/or mitigation policy support, sometimes depending on subsample characteristics moderating the effect [6, 23]. It should be noted that most experimental studies that vary distance to climate change effects to investigate the impact on the willingness to mitigate employ CLT as a framework. While we do not fully agree with the theoretical approach taken, by their design, these studies still provide us with valid empirical evidence about whether distance exerts an effect on the willingness to mitigate. We will recapitulate the findings of the empirical literature so far and point to ambiguities and research gaps that informed our experimental design.

First, let us consider the studies that *did* find a relation between distance to climate change effects and a lower willingness to mitigate. Several correlational studies found an inverse relationship: Greater perceived distance to climate change consequences was linked to less concern [5, 24, 25], lower (stated) behavioral intentions or willingness to act [4, 24, 26, 27], and lower policy support for mitigation [4]. Usually, participants were asked to indicate their agreement to statements such as “Climate change is mostly affecting areas that are far away” [27] or “Climate change will particularly affect me, my family and my friends” [8], indicating how proximate or close they perceived these effects to be. However, while the correlational studies do indicate a link, they do not provide any insights on causal pathways. Establishing causality is a potential strength of the experimental approach. Some of the experimental evidence, in line with the correlational evidence, also suggests that “proximizing” climate change may be a promising strategy to raise awareness and engage people in mitigation behaviors [8, 28]. These studies experimentally altered how close or distant effects of climate change were displayed to the study participants and found that presenting climate change effects as more proximate had a positive effect on concern [8] and (stated willingness to engage in) mitigation behaviors [8, 28, 29].

However, not all studies found that distance reduces the willingness to engage in climate mitigation. Brügger et al.’s [30] results point in the opposite direction: perceptions of both distant and close climate risks were predictive for mitigation policy support and personal mitigation intentions, yet distant risk perception had more explanatory power than close risk perception. This indicates that distance to perceived climate change consequences is *positively* related to the willingness to mitigate. Other experimental studies found no evidence for an effect of distance to adverse climate change consequences on the willingness to engage in mitigation actions or related measures. Displaying climate change effects as more proximate had no effect on attitudes [31], intentions to mitigate [32–34], stated willingness to make a donation to address climate change [35] or policy support [9, 32, 34, 36]. In line with these findings, a study by Kyselá et al. [37] found that agreement to public spending on reducing the risks of climate change did not differ when these risks were said to be reduced nationally as compared to globally.

As a first attempt to understand the ambiguous evidence from existing studies, we delve deeper into differences in study designs, outcome variables and samples.

First, the *manipulations of distance* employed by the studies differed greatly. While using different methods to alter the same object of interest can increase the robustness of results, a deeper look reveals that the distance manipulations altered different aspects of a situation, likely inducing various effects of their own. For instance, in the experiment of Busse and Menzel [33], two subsamples received different questionnaires about environmental problems, one referring to Germany and one referring to a developing country of the respondents’ choice. The authors argue that this would imply a heightened sense of socio-spatial distance. However, the status of a developing country could have effects on its own, as people living in developing countries are perceived as already being in a vulnerable situation, which may induce an other-regarding motivation [38]. This could counter the effect of distance and hence explain the null results in Busse and Menzel’s [33] study. Likewise, Spence et al. [27] found that people show greater concern and mitigation behaviors when they perceive climate change to have an adverse impact on developing countries. Schuldt et al. [39] noticed in this context that some studies did not only vary distance, but also the (severity of) impacts [e.g. mountain pine beetle infestations vs. polar ice melting– 29], which makes it unclear what caused the experimental effects.

Second, studies differ in the measures used to capture *mitigation actions*, and results may be specific to these actions. Those studies finding that distance reduced mitigation action mostly employed stated behaviors or concern as outcome variables so that the findings may be limited to these measures. It is well established that a gap exists between stated and observed behavioral variables (attitude-behavior or value-action gap [e.g. 40]). A replication with observed and costly behavioral measures would thus be important to probe the robustness of the results.

Lastly, various studies have shown that *subsample characteristics* moderate the effect of distance [6, 7]. For instance, political ideology has shown to be a moderating factor to people’s response to the distance treatments, mostly but not always showing that conservatives respond more strongly to a more proximal display of effects [41–43]. Other studies found that previous climate change beliefs of the participants moderated the effect of distance [44]. Hence, there is evidence that different people react differently to the same manipulation and thus, different samples, may influence the outcomes. We are not aware of any experimental studies investigating the role of racism in this context.

For those studies that did find a relation between distance and mitigation actions, the literature so far has not provided definite answers on *how* this effect occurs. For instance, it is unclear whether it is the spatial or social dimension of distance that is responsible for the effect. While Singh et al. [5] found that spatial distance was linked to concern, Gubler et al. [25] did not find evidence of an effect of spatial distance, but rather of social distance. Stanley et al. [24], on the other hand, found support for effects of both distance dimensions. In fact, both dimensions can go hand in hand. For example, a person who grows up and lives in a spatially distant country may also be perceived as being socially far due to a different socio-cultural background. It has long been argued that being similar in terms of socio-cultural background or other relevant features contributes to the perception of social distance [45]. Past studies [e.g. 46] have also shown that the perceptions of spatial and social distance are linked to each other on a cognitive level [which is also a central assumption of CLT—10]. Consequently, in many of the empirical studies, both the spatial and social dimension are varied simultaneously in one treatment, making it impossible to distinguish between the effects of each dimension. For example, presenting climate change effects to either hit the UK or Bangladesh for a UK sample introduces heightened spatial *and* social distance [28]. Accordingly, some studies directly call it ‘socio-spatial distance’ [33].

Studies from the social identity literature have investigated the link between the social and the spatial distance dimension more closely. Several studies suggest that building a global identity–in addition to more regional identities–may be a possibility to overcome distance. There seems to be a correlation between having a global identity and the willingness to preserve nature and the environment [47–49]. For instance, Loy and Reese [49] found that having of a global identity was correlated with a greater willingness to accept mitigation policies. Intergroup contact seems to foster global identity formation. Since spatial distance makes this contact more difficult, it hampers the development of shared social identities.

Adding to the complexity of the experimental results, distance may come with different effects on its own. It seems that spatial distance influences the perception of severity of climate change effects. Several studies, including large-scale cross-cultural studies, suggest that greater distance is linked to the perception of lower environmental quality in general and of greater severity of adverse environmental effects [31, 50, 51]. The direction of this link is, however, challenged by the finding of Zhang et al. [52], who found that water pollution was judged as more severe by the Chinese study participants, when it was presented to affect their local area or people in remote China than when people living on a fictional distant island were affected. It may be that either the fictional character of the island or the type of environmental problem also influenced the severity assessment. For instance, when a particular environmental problem is locally salient–as in the case of water pollution in China [53]–this may break with the general pattern of distant impacts being perceived as more severe. In any case, if perception of severity is coupled with distance, this would likely drive engagement in pro-environmental behaviors and thus come with its own behavioral effect.

At the same time information about more distant effects becomes less personally relevant [46] and people feel less powerful and less responsible to counteract them [54]. As personal responsibility and the feeling of self-efficacy are necessary for people to take action [55], these effects induced by distance may in the end reduce the actual willingness to take action. Moreover, studies centering around social identity theory have found that ingroup sources of information are perceived as more trustworthy and become more influential for action [56]. Studies have also shown that criticism brought forward by outgroup members is met with greater defensiveness [57]–in the case of climate change, reporting about adverse climate effects may be interpreted as a criticism of consumption and production patterns of the Global North, making listeners from the Global North more reluctant to consider this information for action. Hence, if people learn about adverse climate change effects, it is likely to make a difference who is sharing this perspective, namely if it is member of an ingroup or outgroup. Even worse, if intergroup bias exists and outgroup members are distrusted–be it through dissimilarity or devaluation–it becomes more unlikely that consensual solutions are developed [53]. Thus, null results on the impact of distance may be the product of two or more simultaneous and counteracting effects.

In sum, we still do not have solid knowledge of whether, for whom and how distance to climate change effects diminishes the willingness to take mitigation actions. With our study, we aim to contribute to the further understanding of this complex matter.

### The present research

Given the described research gaps, the present study asks: Is there an effect of distance to people negatively affected by climate change on the willingness to engage in costly/observable mitigation actions (RQ1)? Second, and given that we find an effect, we ask: Is it the spatial or social dimension of distance that drives the effect (RQ2)? Third, we examine whether a form of environmental racism may exist regarding the willingness to engage in mitigation actions. We ask: Does racism moderate the effect of distance on the willingness to engage in mitigation actions (RQ3)?

To answer these questions, we conducted an online experiment with a German sample where we presented an interview with a person adversely affected by climate change-induced floods. The text of the interview was identical, but varied name and residence of the affected person (Paul Weber in Germany vs. Samudra Sudarshan in India) in order to induce a variation in distance. To capture the effect of such distance on the willingness to engage in climate mitigation, we used three different measures of mitigation actions–(1) actually donating money, (2) willingness to sign a petition and (3) approving of mitigation polices. The intention of these different outcome measures was to cover varying degrees of costs (low-cost vs. high-cost behavior) and different ways to measure the variables (observed vs. stated behavior).

India as an emerging economy was used as example to reduce possible effects linked to the status of a developing country, such as e.g. Bangladesh. However, people may still associate a different perception of need to a person in Germany versus India, which in turn may influence the mitigation decisions. In the post-experimental questionnaire, we thus elicited expected government support to probe our results against effects that may come with an altered perception of need.

Based on outgroup derogation and potentially environmental racism, we expected that people living in Germany will be less willing to engage in costly mitigation actions when Samudra Sudarshan living in India is affected by adverse consequences of climate change as compared to when Paul Weber living in Germany is affected. We tested this claim as our main hypothesis (preregistered at AsPredicted #38798).

To differentiate between the effects of the spatial and social dimension of distance, we implemented a complementary treatment condition (Samudra Sudarshan in Germany). As the findings of the literature differ on whether it is the social or spatial dimension that accounts for the behavioral effects of distance, we examine this question in a more exploratory analysis without a clear hypothesis.

Last, we measured racist attitudes to probe for evidence of environmental racism. We hypothesized that people with strong racist attitudes would react differently to the distance treatments than people with low/medium racist attitudes. In particular, we expected that a negative effect of distance would be stronger for this subgroup (i.e. we expected to find an interaction effect between racism and the distance treatments).

The remainder of this paper is structured as follows: The second section presents the methodological details of our experiment, giving information about our sample, procedure and operationalization of the concepts looked at. The third section presents the results of our study, including a more in-depth analysis of how effects might unfold. We discuss our findings with respect to the theoretically derived expectations and embed them into the existing literature. The final section concludes by drawing implications for research and policy.

## 2. Materials and method

### Overview

We conducted a survey-embedded online experiment on the Recruitment Platform Clickworker with a German participant pool. The study was run in April 2020. The study protocol was approved by the LaER Ethics Committee of Osnabrück University before running the experiment. The full data set and the instructions can be found here: https://osf.io/zvq9c/?view_only=079950853c5244e0b5c0541b096f2367.

### Participants

450 participants in total were recruited, 150 participants for each of the three treatment conditions. Participants needed to be German residents and be fluent in German to be eligible for the participant pool. A lump sum of 5€ was paid for participation. In addition, participants could receive 0 to 5€, depending on a donation decision they took in the experiment. The payment was framed as a remuneration of 10€ for participation, of which a proportion could be donated to a climate mitigation NGO (the donation constituting one of our three measurement variables).

### Sample description

The age of participants ranged from 18 to 74, with a mean age at 34.75 years. 47.78% of participants indicated to be female, 51.17% to be male, 0.26% to be diverse. Disposable income ranged from the lowest offered category (250€ or less) to the highest (4000€ or more), with a mean of 1702€. 18.54% of participants reported having a migration background (none from India). 18.28% of the respondents declared having experienced flooding themselves in the past.

### Procedure and materials

All participants read short texts providing general information about climate change and its effect to increase the flood intensity as well as the likelihood of heavy flood events. Two attention check items were used to assess whether participants carefully read the text. After this, participants were presented with a first-person report about a flooding event. In the form of an interview, the person described the damage caused by the flooding and emotions associated with the experience. Participants of all treatment conditions hence read the exact same text so that climate change effects were held constant. The interview was assembled from various real interviews conducted with people affected by floods. Afterwards, participants were asked to write a short newspaper article about what happened. This was a further measure to make sure that participants attentively read the manipulation text. Moreover, as a newspaper article requires to answer the questions to whom and where the floods occurred, the task helped to reinforce the saliency of these two distance dimensions.

### Treatment operationalization

**Fig 1:**
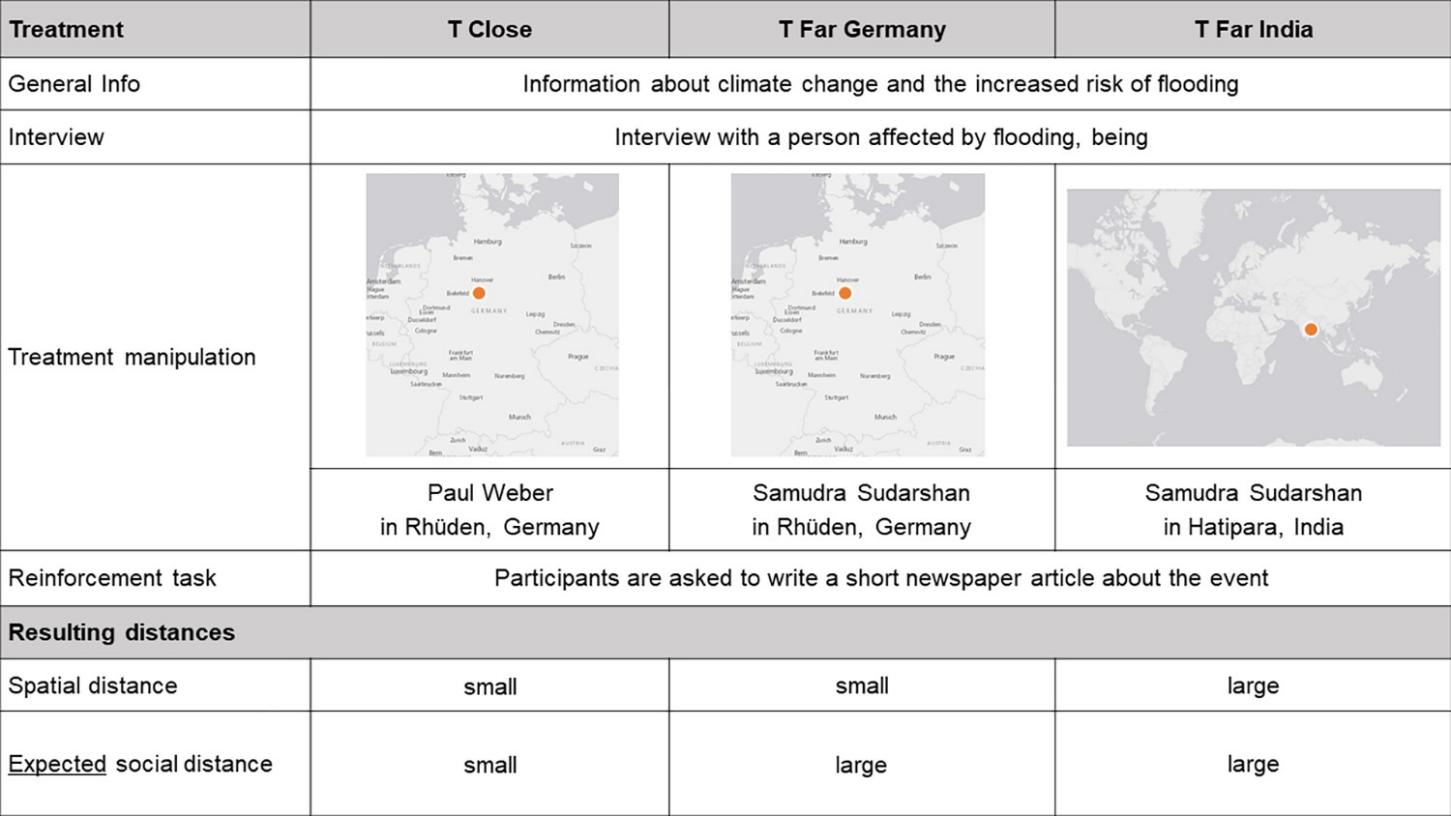
Treatment operationalization. The figure shows how the distance treatments were embedded in the overall survey for the three conditions T_Close_, T_Far Germany_ and T_Far India_, as well as the resulting distances. (Reprinted from USGS National Map Viewer under a CC BY 4.0 license (2022): https://apps.nationalmap.gov/viewer/. The maps are very similar but not identical to the map shown to the participants and therefore only for illustrative purposes).

We manipulated who was affected by the flooding event by altering the name and location of residence of the interviewed person. As we worked with a German participant pool, we employed one condition T_Close_ where the affected person lived in Germany in a small town called Rhüden and who had a name of German origin (“Paul Weber”). As our counter scenario T_Far India_, we used a condition where the affected person lived in India in a similar-sized town called Hatipara and who had a name of Indian origin (“Samudra Sudarshan”). We chose an Indian example since India represents a country of the Global South that is indeed particularly affected by climate change induced floods [58]. Further, India is an emerging economy so the specific effects induced by the status of a developing country as suggested by some studies [27] were likely kept small. To disentangle the effects of spatial and social distance, we conducted an additional scenario T_Far Germany_, where the affected person lived in Germany and had a name of Indian origin (“Samudra Sudarshan”). Fig 1 displays the treatment operationalization and the resulting changes in distance. In combination with the changes in names and place of residence, the geographic maps were used to strengthen the treatment manipulation.

### Manipulation check

To test whether our treatments were salient to the participants, we asked them about the name and residency of the person affected by flooding at the end of the questionnaire via two multiple choice questions. As for the name, almost all participants correctly identified the name (100% in T_Close_, 99.19% in T_Far Germany_ and 100% in T_Far India_). Also for the residence question, correct answers were satisfyingly high (98.51% in T_Close_, 98.37% in T_Far Germany_ and 96.03% in T_Far India_). In T_Far India_, 3.17% answered Iran instead of India, so the effect of distance should still be similar. We can conclude that our treatment manipulations were salient to the participants.

While saliency of the residence can be regarded as objectively inducing spatial distance, the perception of social distance is more subjective. Hence, we further asked participants directly after the treatment to state their agreement on a seven-point Likert Scale (1 = *completely disagree*, 7 = *completely agree*) to the statement “Paul Weber/Samudra Sudarshan and I belong to the same social group” (name according to the treatment participants were allocated to). It is possible that participants answered the item differently according to how they interpreted the term ‘social group’. Still, we found that varying the name and the place of residence of the interviewed person changed the perception of group belonging as expected: The social group score was highest in T_Close_ (M = 3.16, SD = 1.44), lowest in T_Far Gobal_ (M = 1.96, SD = 1.28), and somewhere in-between in T_Far Germany_ (M = 2.77, SD = 1.44). Performing a Kruskal-Wallis test revealed that the differences were significant (*χ*^2^(2) = 43.92, p = 0.0001).

### Operationalization of mitigation actions

Our dependent variable (DV) is willingness to engage in mitigation actions. We operationalized this willingness in several ways to increase the robustness of our results and to examine whether differences exist depending on the type of mitigation measure.

First, we examined whether the distance treatment affected the *willingness to give up own scarce resources* to have a measure of costly and observable behavior. Participants were asked if they wanted to donate parts of their participation remuneration to the NGO atmosfair, which finances CO_2_ offsetting projects (DV1: donation). Participants could donate 0 to 5€ (in steps of 0.50€).

Second, to include an observable but less costly behavior, we assessed whether participants were willing to leave their email address to receive a link to a *petition aimed at climate protection* (DV2: petition). Due to privacy constraints, we could not assess whether they actually signed the petition, but assume that leaving us with their private data and being willing to engage with the topic after the study can be understood as being willing to allocate time and attention for safeguarding the climate.

Third, we measured approval of concrete structural change, by asking participants to indicate their degree of (dis)approval for the introduction of each of a total of 12 *costly political measures* in Germany that are discussed in the context of climate mitigation (DV3: policy approval). The measures include, for instance, higher CO_2_ taxes, a ban of domestic flights or speed limitations on the highway. All measures were briefly explained. On a 5-point scale, participants could indicate whether they *completely disapprove* (-2) to *completely approve* (+2) the introduction. From all items, an average was built to show overall (dis)approval. More details on the dependent variables are provided in the S1 Text.

### Further elicited data

Besides the demographic characteristics of participants (age, income, sex, migration background) and previous flooding experience, we elicited additional data for more in-depth analysis of possible treatment effects.

#### Racism

We elicited racist attitudes building an average of three items taken from GESIS [59]. Participants were asked to rate their agreement to three statements on a 7-point scale (1 = *not at all*, 7 = *fully*). The statements read “I appreciate the diversity of lifestyles, cultures and religions in Germany”, “The foreigners living here threaten our security” and “Whites are rightly leaders in the world” (reverse coding). The three items were part of a battery of other political statements so participants could not easily detect that racism was our main interest.

#### Perception of own affectedness

After the treatments, survey participants were asked to indicate on a seven-point scale to what extent they felt affected by climate change themselves (1 = *not at all*, 7 = *fully*).

#### Government support

Right after being informed about the name and residence of the person being affected by flooding, but before reading the interview, participants were asked to indicate their agreement to the statement “I assume that those affected in Rhüden/Hatipara will receive government support” on a 7-point scale (1 = *not at all*, 7 = *fully*). With government support, we mean, for instance, financial compensation to home owners and help with the rebuilding of infrastructure. In that sense, government support is a proxy of a well-functioning state infrastructure and national wealth and can thus be taken as a control variable for the potential effects of need induced by the status of a developing country.

### Data exclusion

Online experiments need rigorous data quality control as the attention of participants cannot be controlled for by the experimenter [60]. To ensure high data quality, we decided ex ante (see preregistration protocol) to exclude the data of participants who

- (i) had very short answering times (less than half of the average answering times),
- (ii) indicated they gave “meaningless responses” frequently or sometimes, or
- (iii) failed both attention check questions we asked after the information texts.

Following these criteria, our final data set consisted of 383 observations– 134 in T_Close_, 123 in T_Far Germany_ and 126 in T_Far India_. To control for a potential bias in drop out, we estimated the dropout rates on basis of the treatment groups. No significant differences could be detected. We further conducted balancing tests among the treatment groups for age, income, sex, racism, migration background and own flooding experience, which confirmed that the randomization resulted in a balanced sample (see S1 Table for details).

### Analysis

We used non-parametric tests (Chi2 and Mann-Whitney-U tests, Bonferroni-corrected) to assess if treatments had an effect on the willingness to engage in mitigation actions. To test for robustness, mediation channels and moderation effects, we used regression analysis including various control variables. Depending on the type of the dependent variable–numeric or binary, we either used linear regression (policy approval) or a probit regression model (petition, donation), respectively. We report results as significant when α ≤5%. The descriptive statistics of our data can be found in S2 Table.

## 3. Results and discussion

### RQ1: Is there an effect of distance to people negatively affected by climate change on the willingness to engage in costly/observable mitigation actions?

Our first research question asked *whether* distance to those adversely affected by climate change would influence the willingness to engage in mitigation actions. We hypothesized that people living in the Global North would be less willing to engage in mitigation actions when it was a person living in the Global South who was affected by the adverse consequences of climate change, as compared to a person living in the Global North. To test this hypothesis, we compared the treatments T_Close_ and T_Far India_ with regards to the participants’ mitigation decisions. In this subsection, we discuss results with respect to the three different mitigation measures we applied in our study as dependent variables.

**Fig 2:**
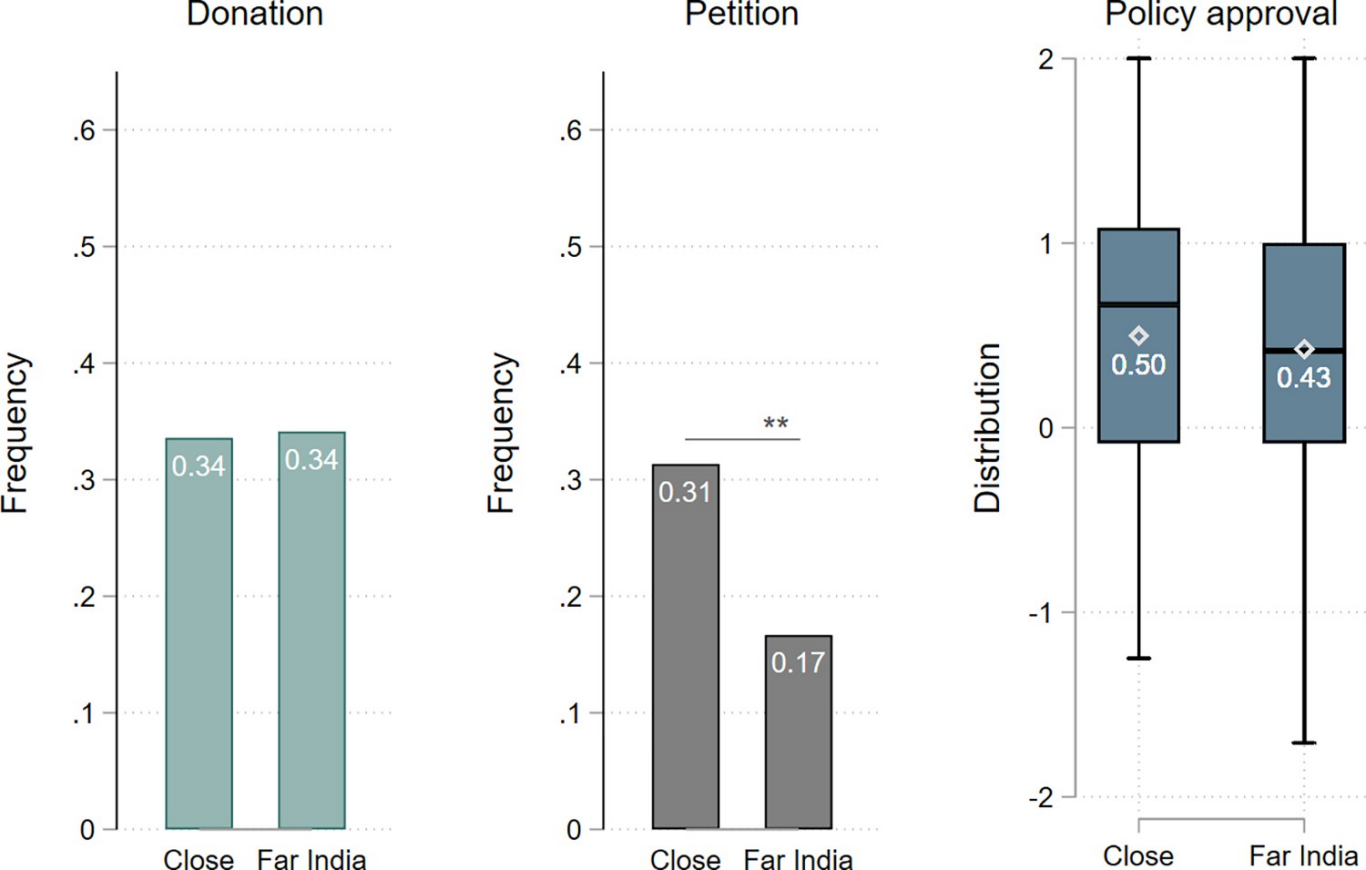
Mitigation actions, comparing treatments Close and Far India. For the variables donation and petition, the scale shows the share of people choosing to make a donation or to give their email address to sign the petition (1 corresponding to 100%). For policy approval, the scale shows the average agreement to 12 policy measures (2 = full approval to -2 = full disapproval).

Fig 2 shows the bar graphs for all three dependent variables, comparing T_Close_ and T_Far India_. For the statistical analysis we employed Chi2- and Mann-Whitney-U-tests. We found support for our hypothesis in one of the three mitigation measures, namely for the willingness to sign a petition for climate protection (T_Far India_ vs. T_Close_: *χ*^2^(1) = 7.62, p = 0.018). When a person in Germany with a name of German origin reported being affected by climate change induced floods, 31% of the survey respondents from Germany were willing to give their email address to sign a petition for more climate protection. When instead a person in India with a name of Indian origin told the exact same story, this share was almost cut in half to 17% of the respondents. This result supports the hypothesis that distance negatively affects the willingness to engage in climate protection. For the other mitigation measures (donation and policy approval), however, the hypothesis could not be sustained (*χ*^2^(1) = 0.009, p = 1.00 and Z = 1.51, p = 0.20, respectively). For the donation variable, results were the same when looking at it as a binary variable (yes-no) or as the amount donated, so we use the binary form here and report the more elaborate analysis in the S3 Table.

We probed the robustness of our results applying multivariate regression analysis, and controlling for sociodemographic characteristics as well as own flooding experience by the participants. Including these controls reduced the number of observations due to missing responses for these variables (here: n = 215). Table 1 shows the corresponding estimation results. Models 1 and 2 present the results of probit regressions estimating the likelihood of making a donation (Model 1) and of signing a petition (Model 2), while Model 3 shows linear regression (OLS) results for the policy approval measure. In the supporting information, we provide the detailed regression table with all control variables listed separately (S4 Table). Models 4–6 include, in addition, the data from the additional treatment T_Far Germany_ and a corresponding treatment dummy variable.

**Table 1:** Regression results for treatment effects.

	(1)	(2)	(3)	(4)	(5)	(6)
	Donation	Petition	Policy approval	Donation	Petition	Policy approval
**Far India**	-0.0695	-0.500*	-0.0715	-0.0782	-0.487*	-0.0668
	(0.179)	(0.199)	(0.109)	(0.178)	(0.196)	(0.105)
**Far Germany**				0.229	-0.018	0.127
				(0.175)	(0.181)	(0.105)
Constant	-0.288	-1.120**	0.360	-0.417	-1.095***	0.337*
	(0.337)	(0.367)	(0.205)	(0.278)	(0.296)	(0.165)
N	215	215	215	315	315	315
Controls included	yes	Yes	yes	yes	yes	yes
**p: Far India = Far Germany**				0.093	0.020	0.076

This table shows the estimation results from regressing the impact of the treatment conditions Far India and Far Germany on the willingness to participate in mitigation actions, measured by three mitigation variables: Donation, Petition, and Policy approval. Model (1)–(2) and (4)–(5) show the coefficients for probit regressions on the likelihood of making a donation or signing the petition. Models (3) and (6) are based on an ordinary least squares regression model that estimates the effect of the treatment conditions on the average approval of 12 realistic policy measures for climate protection in Germany. Controls included are sociodemographic characteristics such as age, gender, disposable income, migration background as well as own flood experience. Standard errors are indicated in parentheses. The symbols *, **, *** indicates p<0.05, p<0.01, and p<0.001, respectively. Full sample size n = 383.

The regression analysis confirms the results obtained by the non-parametric tests: the negative effect of T_Far India_ as compared to T_Close_ on the willingness to sign a petition is statistically significant and robust (p = 0.012 in Model 2 and p = 0.013 in Model 5). Results for the donation and policy approval remain non-significant. As a further robustness check, we added expected government support as a control variable for all three dependent measures and found that it did not impact our findings (S4 Table). Government support itself was not a significant predictor. So, whether or not people expected the state to help the person affected by flooding did not influence our results. We may interpret this in a sense that a differing perception of need, as associated for instance with the socio-economic status of a country, did not affect the mitigation decisions of the participants as indicated by previous studies. However, we acknowledge that our government support variable is not a perfect proxy for this perception, as it was not specified how significant this support would be and further socio-economic factors may add to it, so we recommend that these links are investigated with more scrutiny in future studies.

In sum, our findings that T_Far India_ reduced the willingness to sign a petition while there was no effect on the willingness to make a donation or approve of mitigation policies can be regarded as robust. To further test these results, future studies could also control for other factors like previous climate change beliefs or knowledge, as some studies have found that concern and novelty of information could moderate the effects [42, 44].

Taken together, our results provide some evidence for the existence of a negative effect of distance to adverse climate change effects on the willingness to engage in mitigation actions. Yet, our findings also indicate that this effect is not uniform across our different outcome variables capturing mitigation actions: While for the petition, we found an effect of varying the distance to the person adversely affected by climate change, we did not find any such effects for donating money or policy support. This dependence of results on the specific mitigation actions may explain the mixed evidence found in previous experimental studies. But what are the reasons for these different findings?

We employed as outcome variables mitigation measures that could be distinguished in terms of the personal costs or constraints involved (costly vs. low-cost) and with regards to how the measure was elicited (stated vs. observed). Not surprisingly, the costliness of the decision matters for the course of action, as we can see when comparing our two observed variables, donation and petition. Although signing a petition implies spending time and attention to an issue, it is a relatively easy behavior, which does not involve any pecuniary costs or personal disadvantages. This is particularly so, since in our study we did not measure actual signing of the petition, but took the provision of an email address for being sent more information on the petition as a proxy. In comparison, donating part of one’s remuneration involves an immediate monetary cost. Thus, it is intuitive that our treatment had an effect on the low-cost behavior (signing the petition) while there was no impact on the costly behavior (donating money).

However, if immediate costs were the only decisive factor, one may have expected that for our policy support measure, we would observe the strongest treatment effect. Answering the policy support questions neither implied immediate costs nor had real-life consequences for the participants. Still, our treatment did not impact policy support. Possibly, this can be explained by the nature and concreteness of the suggested policy measures. We only chose costly or restrictive political measures, such as higher taxes or a mandatory Veggy day. All measures were policy options discussed in the actual public debate on how to achieve climate mitigation. As such, they likely succeeded to trigger realistic consideration by the study participants, implying high costs if the measures were actually implemented. Moreover, approval or disapproval of the policy measures could be linked to more encompassing political or partisan identities and values, which are known to be constant at least in the short-term [61, 62].

Our result is in line with several experimental studies that did not find an effect of experimentally varying distance on policy approval [9, 32, 34, 39]. However, several other studies did report an impact of distance on policy approval [e.g. 41, 42], at least for subgroups of participants [43, 46]. The difference could be explained by the non-costly nature of the policies used by these latter studies (e.g. tax rebates–[42]), the political ideology dominant in the sample (e.g. more conservatives responding more strongly to the distance treatments [41]), the administrative level of the policy (different response patterns emerged for subnational to national to international policies [41]), the time of implementation of the policy (inducing another dimension of distance [63]) and the type of environmental risk addressed by the policies (different response patterns depending on which environmental problem was sought to be mitigated by the policies [37]).

In sum, we found that increasing the distance to the person adversely affected by climate change reduced our observed but low-cost mitigation action, while it had no effect on the observed costlier action or stated support for costly policy measures for mitigation.

### RQ2: Is it the spatial or social dimension of distance that drives the effect?

We found robust evidence that people in Germany were less willing to sign a petition for more climate protection, when a person in India with a name of Indian origin was affected as compared to a person in Germany with a name of German origin. Based on this finding, we were interested in assessing whether the geographical distance or the social distance to people with a different socio-cultural background was responsible for the effect. For this purpose, we used our third treatment condition TFar Germany−in which the interviewed person was someone living in Germany with a name of Indian origin. We included observations of this additional treatment group in the regression estimations of Table 1 (Models 4–6). The resulting sample size for these regressions including controls was 315.

Comparing T_Close_ with T_Far Germany_, there is no effect on our outcome variable petition (Model 5 in Table 1). Between these conditions only the name changed, while the residence was kept constant. The result thus suggests that social distance did not induce the effect. By contrast, comparing T_Far Germany_ and T_Far India_, we found that changing only the country where the climate change induced floods occurred from Germany to India significantly affected the willingness to sign the petition (*χ*^2^(1) = 5.42, p = 0.02): The willingness was lower when the affected person (Samudra Sudarshan) was located in India as opposed to Germany. Hence, we take this as a first indication that spatial distance significantly contributes to a lower willingness to engage in mitigation actions, while social distance seems to have no effect.

However, as we saw when analyzing our manipulation check (the social group score), changing the residency from T_Far Germany_ to T_Far India_ also led to a change in social distance. Thus, it may be that the behavioral difference between T_Far Germany_ and T_Far India_ we reported above and assumed to be stemming from the difference in spatial distance may also partly be due to the heightened perception of social distance that came with the altered residency. Hence, we opted for a more elaborate analysis. Table 2 presents the results of multivariate regression analyses probing whether the treatments altered the perceived social distance (Model 1) and whether this mediated the treatment effect (Model 2–4).

**Table 2:** Regression results for social group belonging as possible mediation pathway.

	Perceived social group belonging			
	(1)	(2)	(3)	(4)
	Social group	Petition	Petition	Petition
**Far India**	-1.298***	-0.487*		-0.457*
	(0.194)	(0.196)		(0.208)
**Far Germany**	-0.471*	-0.018		-0.0059
	(0.194)	(0.181)		(0.183)
**Social group belonging**			0.0661	0.0241
			(0.052)	(0.055)
Constant	3.332***	-1.095***	-1.423***	-1.176***
	(0.304)	(0.296)	(0.316)	(0.349)
N	315	315	315	315
Controls included	yes	Yes	yes	yes
p: Far India = Far Germ	<0.001	0.020		0.028

Regression models presented in this table examine whether an altered feeling of social distance mediated the treatment effect on the willingness to sign the petition. Model (1) is based on ordinary least square regression models, examining whether the social group perception was influenced by the treatments. Model (2)-(4) estimate the likelihood of signing the petition, employing a probit model. Model (2) is the same as Model (5) of Table 1. Model (3) assesses the impact of social group belonging on the willingness to sign the petition. Model (4) then includes social group belonging in the original estimation of Model (2) and assesses whether social group belonging mediated the effect of the treatment conditions. Controls included are sociodemographic characteristics such as age, gender, disposable income, migration background, as well as own flood experience.

The symbols *, **, *** indicate significance for p<0.05, p<0.01, and p<0.001, respectively. Full sample size n = 383.

The regression in Model 1 replicates the finding from the analysis of the manipulation check: our treatments induced different perceptions of social distance (p = 0.015 for T_Far Germany_ and p<0.001 for T_Far India_). Conducting an F-test probing the equality, we see that there also exists a significant difference between T_Far Germany_ and T_Far Global_ (F(1,306) = 17.09, p< 0.001), with T_Far Global_ having the stronger effect.

Model 2 is the same as Model 5 in Table 2, we show it here again to ease comparability. To investigate whether social distance explains our previous results, we evaluated in Model 3 and 4 its predictive power for signing the petition. We found that social distance is not a significant predictor of participants’ willingness to sign the petition (p = 0.20 in Model 3 and p = 0.66 in Model 4). When including both, the proxy for social distance (the social group score) and the one for spatial distance (the treatment dummy for T_Far India_) as predictors in Model 4, the influence of spatial distance continues to be significant (p = 0.03). Thus, while our treatment conditions have affected participants’ perception about the social distance, we can conclude that it was indeed the spatial dimension of distance that lowered participants’ willingness to engage in climate mitigation. Future research could examine whether a different measure of social distance–e.g. perceived similarity–would have yielded the same results.

While we can say from our analysis that spatial distance rather than social distance had an effect, an interesting question is what it is about the spatial distance to climate change effects that reduces people’s willingness to act. Exemplarily, we looked at how spatial distance may influence the *perception of personal affectedness*, which could in turn affect the willingness to act [27]. We found that perceived personal affectedness was reduced when the country changed from Germany to India. Personal affectedness also predicted the willingness to sign a petition. However, it did not mediate the effect of spatial distance (please refer to S5 Table for statistical details). Other possible mediation pathways to explain why spatial distance reduces the willingness to mitigate, as suggested by other studies [46, 54], could be that spatial distance lowers personal relevance or personal responsibility to act. While our study did not permit testing for these pathways, these could be relevant extensions for future studies.

### RQ3: Does racism moderate the effect of distance on the willingness to engage in mitigation actions?

Lastly, we explored whether environmental racism moderates the treatment effect. As moderation may also take place with respect to those outcome variables, for which we did not find an effect at the level of our total sample, we looked at all three mitigation variables again.

For the combined racism item (see section 2), the average score on the 7-point-scale was relatively low (M = 2.22, SD = 1.15). Additionally, it was an item that a considerable amount of people (18 participants) did not answer at all. Both observations can be taken as a hint for social desirability bias playing a role in the answers on these items. To counter this bias, we looked at the extremes. Specifically, we constructed a dummy variable for high racism, indicating whether an individual score was above the 90^th^ percentile (46 participants fell into this category).

We checked if the treatments interact with environmental racism by including an interaction term of high racism and each treatment variable in the regression models that included the sociodemographic controls (S6 Table). The interaction terms were not significant for donating money (High Racism*T_Far Germany_: b = -0.68, p = 0.37; High Racism*T_Far India_: b = -0.61, p = 0.39) nor for signing the petition (High Racism*T_Far Germany_: b = 0.16, p = 0.81; High Racism*T_Far India_: b = -0.39, p = 0.72). For policy approval, we found a significant interaction with the T_Far Germany_ treatment (b = -0.77, p = 0.03), yet none for T_Far India_ (b = -0.34, p = 0.30).

**Fig 3:**
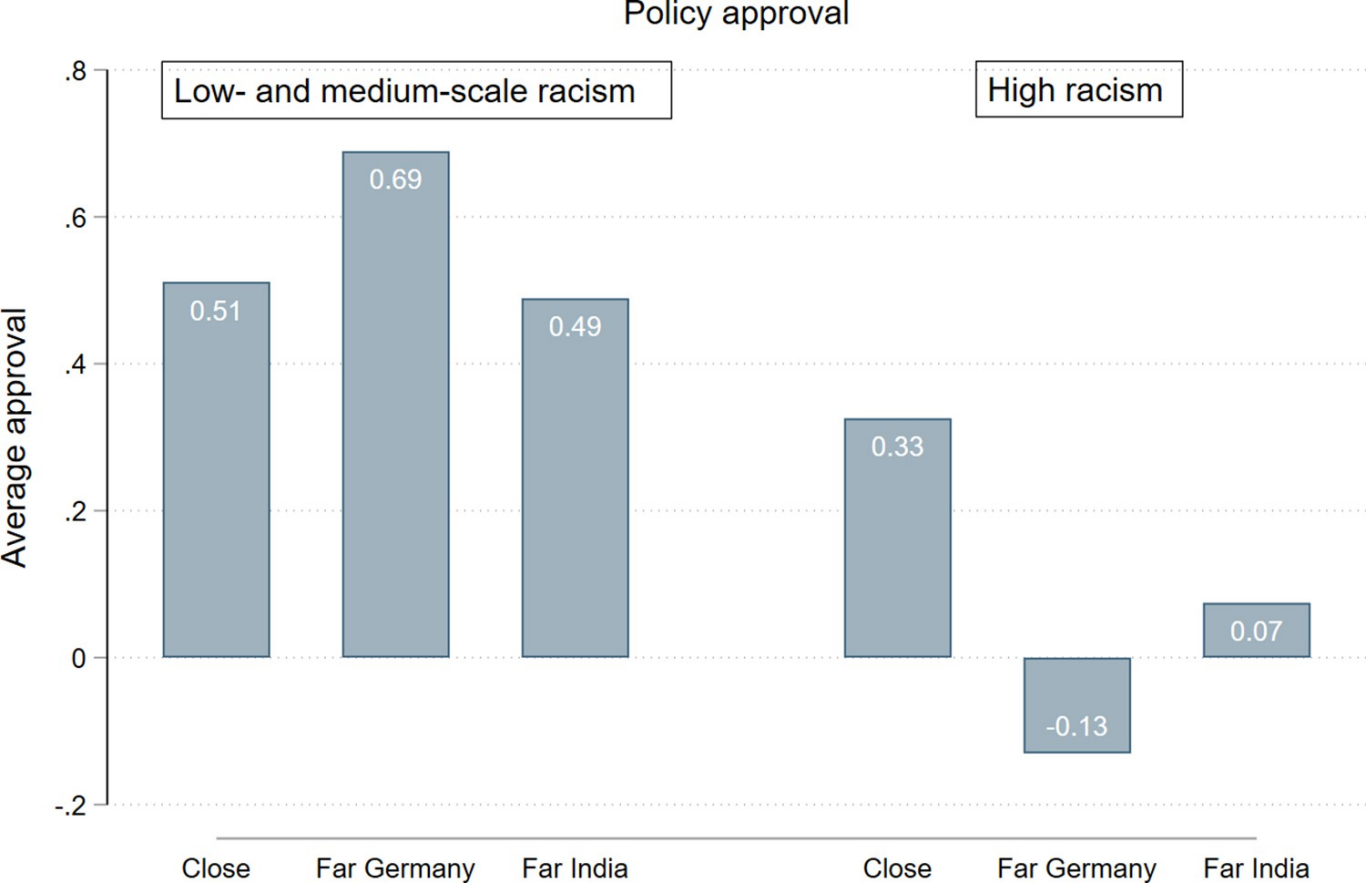
Different reaction patterns (policy approval) to the treatments depending on racist attitudes. The scale shows the average agreement to 12 policy measures (2 = full approval to -2 = full disapproval).

Fig 3 shows the interaction effect for policy approval graphically. We see that the reaction patterns for people scoring high on racism are different to those with low and medium scores. While this latter group does not seem to be particularly responsive when the country changed from Germany to India, we observe a drop in policy support for those scoring high on racism. Moreover, in the T_Far Germany_ condition, there seems to be even a reversing effect: Participants with strong racist attitudes rejected mitigation policies when the name changed from Paul Weber to Samudra Sudarshan while the location of the flooding remained to be Germany. Participants likely attributed a migration background and darker skin color to the name, which we assume was responsible for the moderating effect of racism.

The graph also reveals that people scoring high on racism in general show less support for mitigation policies. Indeed, racism itself is a strong and significant negative predictor for all three mitigation variables (donation: b = -0.26, p = 0.001; petition: b = -0.18, p = 0.015; policy support: b = -0.21, p<0.000, controls included–S7 Table). Overall, concerning the question if racism moderates the effect of distance on the willingness to engage in mitigation actions, our results provide only weak evidence. We detected this effect only for policy approval and when the person affected was located in the same country as the respondent. While the latter finding might seem counterintuitive at first sight, it is not surprising that racist attitudes come out particularly strongly when those derogated by racism are close to them. As for only finding a significant moderation effect for policy approval and not for the donation and petition, it is not clear why this is the case. Following our argumentation from above, we would have expected to see main or moderation effects for the same variable that was most responsive to our treatments, namely the petition as a low-cost action. It could be, however, that our study lacks explanatory power on the issue of environmental racism because social desirability bias in the responses on racism led to small numbers of observations with high racism. Further research is needed to follow up on these tentative results.

## 4. Conclusions

We examined whether the distance to people negatively affected by climate change plays a role for the willingness to engage in costly and/or observable mitigation actions. We found that the willingness to sign a petition for more climate protection was significantly reduced when a person in India with a name of Indian origin was affected by flooding as compared to a person in Germany with a name of German origin. For donating money to CO_2_ offsetting as well as for support of mitigation policies, there was no such effect. We argue that this is because these two behaviors involve higher (potential) costs for the respondents as compared to the willingness to sign a petition. The cost aspect seemed to matter more than whether the behavior was observed or stated. Our results support the idea that the ambiguous findings of prior studies can, at least partly, be explained by the different costs of the mitigation actions that were measured as outcome variables. In sum, our experimental results suggest that there is a negative effect of distance for low-cost measures of mitigation actions.

Moreover, we asked whether the impact of distance could be attributed to the spatial or the social dimension of distance. We did not find evidence that social distance exerted an effect on the willingness to mitigate. Rather, the effect can be attributed to spatial distance to the adverse climate change effects. Hence, we did not find support for the idea that outgroup derogation as suggested by social identity theory would account for an effect of distance on the willingness to mitigate, which we offered as an alternative explanation to the often referred to explanation by CLT. Exploring how spatial distance affected the willingness to mitigate, we found no evidence that the perception of being affected by climate change oneself could explain this effect. Following-up on our results, further studies would be needed to investigate what it is exactly about the spatial dimension of distance that explains its effect. Impacts of spatial distance on personal relevance or personal responsibility to act could be relevant avenues to explore more rigorously.

Finally, we found weak evidence that racism might moderate the effect of distance, even to an extent that seems irrational: People with strong racist attitudes dropped their support for mitigation policies when a person with a name of Indian origin was affected by floods as compared to a person with a name of German origin, both having a residence in Germany. We interpret this as a form of reactance to give up own resources when others are affected who are not valued by those potentially engaging in mitigation efforts. Thus, we take this as tentative evidence for a form of environmental racism that reduces the willingness to mitigate, at least for some, when people who are racialized are most severely affected.

It should be noted that our study was a short-term intervention that only changed a few words to vary proximity and distance, supported by a map to display the location. This was sufficient to alter low-cost mitigation actions. Further research could evaluate whether stronger interventions, e.g. more long-term interventions or interventions using pictures or videos of people, would induce an effect also for more costly mitigation actions. In addition, an interesting avenue for future research is to examine whether our findings can be replicated with samples from different countries in the Global North or Global South. For instance, a future study could apply a slightly adjusted study design to an Indian sample with India as the Close condition and investigate if effects are the same. Future studies could also investigate more systematically alternative mediation pathways for an effect of distance on the willingness to mitigate. In addition, more rigorous studies are needed to test for the existence of environmental racism as we defined it. Using implicit methods [64] to measure racism could help to reduce social desirability bias in the answers and thus provide more definite insights.

In the context of climate change, societies must deal with the remaining challenge how to collectively engage its citizens in mitigation efforts that are both costly and to the benefit of people at a distance. As we lack an understanding of what it is about the spatial dimension that reduced the willingness to mitigate, we cannot deduce any concrete policy recommendations. However, policy-makers should acknowledge that distance to effects can indeed play a role in the willingness to mitigate, at least for low-cost actions. A more general conclusion is this: As the mismatch of need and action is something that happens at the individual level, there is a necessity for political solutions at the collective level, e.g. global agreements with fixed goals and measures–even though these come at their own difficulties.

Regarding environmental racism, if this finding can be reproduced by future research, tailoring communication strategies to meet the racist attitudes of people cannot be the solution. Rather, the challenging question remains how people can care enough to become active to an extent that does justice to the urgency of halting climate change, even when distant others are affected, and how a (global) society can attach value to and care for all humans regardless of origin, skin color etc. While the role of racism for the effect of distance still needs further investigation, racism itself is undoubtedly and strongly linked to a low engagement in mitigation actions in our study. Thus, it seems that achieving climate justice and achieving social justice are transformations that best go hand in hand.

## Supporting information

S1 Text Dependent variables. (DOCX)

S1 Table Balancing table. (DOCX)

S2 Table Descriptive statistics. (DOCX)

S3 Table Complementary analysis for donations—Two-stage estimation. (DOCX)

S4 Table Regression results with additional controls. (DOCX)

S5 Table Regression results for perceived personal affectedness. (DOCX)

S6 Table Regression results for high racism. (DOCX)

S7 Table Racism as predictor for mitigation actions. (DOCX)
