# Supplementary material for: Distance to climate change consequences reduces willingness to engage in low-cost mitigation actions–Results from an experimental online study from Germany
Source: PLoS One. 2023 Apr 5;18(4):e0283190. doi: 10.1371/journal.pone.0283190 (PMC10075397; doi:10.1371/journal.pone.0283190)
Supplement: S1 Text — (DOCX) [file pone.0283190.s001.docx]

## S1 Text. Dependent variables.

**Dependent variables (DV) measuring mitigation actions**

| **DV 1: Donation** | Actual donation of part (0-5€) of the 10€ remuneration to the NGO atmosfair (=1 if donation is provided, = 0 otherwise) |
| --- | --- |
| **DV2: Petition** | Indication of email address for signing a petition for more climate protection (Petition) (=1 if email address is provided, = 0 otherwise) |
| **DV3: Policy approval** | Willingness to approve of 12 mitigation policies to be introduced in Germany (-2 fully against; +2 fully in favor), DV3 is average of approval for all policies |

**DV1: Donation (German original)**

**Wir möchten uns für Ihre Teilnahme bis hierhin bedanken!**

Als Vergütung werden Ihnen 10 Euro über die Plattform Clickworker gutgeschrieben.

Sie können sich entscheiden, Teile dieser Vergütung (maximal 5€) an die Klimaschutzorganisation atmosfair zu spenden.

atmosfair finanziert Klimaschutzprojekte im Bereich Erneuerbare Energien sowie Umweltbildung an Schulen. Die Projekte, die atmosfair unterstützt, sind nach dem höchsten Standard für CO2-Einsparungsprojekte zertifiziert (CDM Gold Standard). 1€ entspricht dabei in etwa einer Kompensation von 40 kg CO2.

Möchten Sie an atmosfair spenden?

- Ja
- Nein

[Wenn Antwort Ja] Wie viel möchten Sie spenden?

Ich spende [Auswahl von 0-5€, in Schritten von 0.50€]

***English translation:***

**We would like to thank you for your participation up to this point!**

As compensation, you will be credited with 10€ via the Clickworker platform.

You can decide to donate parts of this compensation (maximum 5€) to the climate protection organization atmosfair.

atmosfair finances climate protection projects in the field of renewable energies as well as environmental education at schools. The projects atmosfair supports are certified according to the highest standard for CO2 reduction projects (CDM Gold Standard). 1€ is roughly equivalent to offsetting 40 kg of CO2.

Would you like to donate to atmosfair?

- Yes
- No

[If yes] How much do you want to donate?

I donate [selection of 0-5€, in steps of 0.50€]

**DV2: Petition (German original)**

**Möchten Sie mitmachen?**

Wenn Sie den Link zum Unterschreiben einer aktuellen Petition zur Stärkung der Klimaschutzpolitik zugeschickt bekommen möchten, bitte hinterlassen Sie hier Ihre Email-Adresse.

Ihre Email-Adresse wird separat von den restlichen Daten dieses Fragebogens gespeichert, sodass Ihre Anonymität gewahrt bleibt.

- Ja, bitte senden Sie mir den Link per Email zu.
- Nein, danke.

***English translation:***

**Do you want to participate?**

If you would like to receive the link to sign a current petition to strengthen climate protection policy, please leave your email address here.

Your email address will be stored separately from the rest of the data in this questionnaire so that your anonymity is preserved.

- Yes, please send me the link by email.
- No, thank you.

**DV3: Policy approval (German original)**

Nun interessiert uns noch Ihre Einstellung zu unterschiedlichen politischen Maßnahmen und Vorhaben, die im Kontext des Klimawandels diskutiert werden.

Bitte geben Sie an, wie Sie *persönlich* zu einer Umsetzung dieser Vorhaben stehen.

|  | Voll und ganz dagegen  -2 | Eher dagegen  -1 | Unent-schieden  0 | Eher dafür  +1 | Voll und ganz dafür  +2 |
| --- | --- | --- | --- | --- | --- |
| **Verbindliche Festschreibung des 1,5°C-Ziels für Deutschland:**  In einem Klimaschutzgesetz wird die Einhaltung des 1,5°C-Ziels des Pariser Klimaschutzabkommens durch Deutschland festgeschrieben und mit konkreten Überprüfungs- und  Sanktionsmechanismen untersetzt. |  |  |  |  |  |
| **Erhöhung des EU-weiten Minderungsziels von Treibhausgasen:**  Die EU verschärft bis 2030 schrittweise ihr Minderungsziel von Treibhausgasen von 40% auf 55% und schließlich auf 65%. |  |  |  |  |  |
| **Ausstieg aus der Kohleenergie bis 2030 statt 2038:**  Die Deutschland steigt schon 2030 aus der Kohleenergie aus – acht Jahre vor dem für 2038 geplanten Ausstieg, der durch die sog. Kohlekommission verhandelt wurde. |  |  |  |  |  |
| **Abbau der Subventionen fossiler Energien:**  Subventionen von fossilen Energieträgern (Umfang 2017: 46  Mrd. Euro) werden mit einem verbindlichen Zeitplan abgebaut. Dadurch werden voraussichtlich einige Produkte zunächst teurer. |  |  |  |  |  |
| **Verbot von Inlandsflügen:**  Flüge innerhalb von Deutschland werden gesetzlich verboten. |  |  |  |  |  |
| **Einführung eines Tempolimits auf der Autobahn:**  Es wird eine von 120 km/h für den Verkehr auf Autobahnen eingeführt, um den CO2- und Schadstoffausstoß des  Individualverkehrs zu senken. |  |  |  |  |  |
| **Erhöhung der Steuern und Abgabe auf Flugreisen:**  Höhere Steuern und Abgaben auf Flugreisen (Kerosinsteuer,  Luftverkehrsabgabe) erhöhen den Preis für den Flugverkehr. |  |  |  |  |  |
| **Ausstieg aus dem Verbrennungsmotor:**  Es wird gesetzlich geregelt, dass ab 2025 keine neuen  Personenwagen mit Verbrennungsmotor zugelassen werden. |  |  |  |  |  |
| **VeggyDay in öffentlichen Einrichtungen:**  In öffentlichen Einrichtungen wird an einem Tag pro Woche  ausschließlich vegetarisches Essen angeboten, um die  klimaschädigenden Wirkungen des Fleischkonsums zu senken. |  |  |  |  |  |
| **Erhöhung der neuen CO2-Steuer:**  Die neu eingeführte CO2-Steuer wird mit 50€ pro Tonne CO2 (momentan geplant 25€) eingeführt und bis 2025 auf 180€ erhöht (momentan geplant 55€), sodass CO2-intensive Produkte im Vergleich (noch) teurer werden. |  |  |  |  |  |
| **Umverteilung von Vermögen:**  Vermögen wird in Deutschland so umverteilt, dass sich alle Menschen eine klimaverträgliche Lebensweise leisten können. |  |  |  |  |  |
| **Umfassender wirtschaftlicher und gesellschaftlicher**  **Systemwandel:**  Ein umfassender wirtschaftlicher und gesellschaftlicher  Systemwandel wird in Deutschland angestrebt, um eine  klimaverträgliche Lebensweise zu ermöglichen. |  |  |  |  |  |

***English translation:***

We are now interested in your attitude to various political measures and projects that are being discussed in the context of climate change.

Please state how you *personally* feel about the implementation of these measures.

|  | Fully against  -2 | Rather against  -1 | Indifferent  0 | Rather in favour  +1 | Fully in favour  +2 |
| --- | --- | --- | --- | --- | --- |
| **Binding establishment of the 1.5°C target for Germany:**  In a climate protection law, Germany's compliance with the 1.5°C target of the Paris Climate Agreement is stipulated and backed up by concrete monitoring and sanction mechanisms. |  |  |  |  |  |
| **Increasing the EU-wide greenhouse gas reduction target:**  The EU gradually tightens its greenhouse gas reduction target from 40% to 55% and eventually to 65% by 2030. |  |  |  |  |  |
| **Phasing out coal power by 2030 instead of 2038:**  Germany is already phasing out coal energy in 2030 - eight years ahead of the planned phase-out in 2038, which was negotiated by the so-called Coal Commission. |  |  |  |  |  |
| **Reduce fossil fuel subsidies:**  Subsidies of fossil energy sources (volume in 2017: 46 billion euros) are being phased out with a binding timetable. This is expected to make some products more expensive initially. |  |  |  |  |  |
| **Ban on domestic flights:**  Flights within Germany will be banned by law. |  |  |  |  |  |
| **Introduction of a speed limit on the autobahn:**  A speed limit of 120 km/h will be introduced for traffic on highways in order to reduce CO2 and pollutant emissions from individual traffic. |  |  |  |  |  |
| **Increase taxes and levies on air travel:**  Higher taxes and levies on air travel (kerosene tax, air traffic tax) increase the price of air travel. |  |  |  |  |  |
| **Phasing out the internal combustion engine:**  Legislation will stipulate that from 2025 onwards, no new passenger cars with internal combustion engines will be permitted. |  |  |  |  |  |
| **VeggyDay in public institutions:**  In public institutions, one day a week exclusively vegetarian food is served in order to reduce the climate-damaging effects of meat consumption. |  |  |  |  |  |
| **Increase of the new CO2 tax:**  The newly introduced CO2 tax will be introduced at 50€ per ton of CO2 (currently planned 25€) and will be increased to 180€ by 2025 (currently planned 55€), making CO2-intensive products (even) more expensive in comparison. |  |  |  |  |  |
| **Redistribution of wealth:**  Wealth is redistributed in Germany so that all people can afford a climate-friendly lifestyle. |  |  |  |  |  |
| **Comprehensive economic and social system change:**  Comprehensive economic and social system change is being pursued in Germany in order to achieve a climate-friendly way of life. |  |  |  |  |  |
