# Supplementary material for: Distance to climate change consequences reduces willingness to engage in low-cost mitigation actions–Results from an experimental online study from Germany
Source: PLoS One. 2023 Apr 5;18(4):e0283190. doi: 10.1371/journal.pone.0283190 (PMC10075397; doi:10.1371/journal.pone.0283190)
Supplement: S1 Table — (DOCX) [file pone.0283190.s002.docx]

## S2 Table. Balancing table.

|  | **(1)** | | **(2)** | | **(3)** | | **t-test** | **t-test** | **t-test** |
| --- | --- | --- | --- | --- | --- | --- | --- | --- | --- |
|  | **Close** | | **Far Germany** | | **Far India** | | **Difference** | **Difference** | **Difference** |
| **Variable** | **N** | **Mean/SE** | **N** | **Mean/SE** | **N** | **Mean/SE** | **(1)-(2)** | **(1)-(3)** | **(2)-(3)** |
| **Age** | 130 | 35.977 | 116 | 34.310 | 123 | 33.854 | 1.667 | 2.123 | 0.457 |
|  |  | [1.145] |  | [1.157] |  | [1.039] |  |  |  |
| **Gender (not male)** | 134 | 1.590 | 123 | 1.520 | 126 | 1.508 | 0.069 | 0.082 | 0.012 |
|  |  | [0.048] |  | [0.049] |  | [0.049] |  |  |  |
| **Disposable income (in EUR)** | 118 | 1,740.466 | 105 | 1,617.857 | 103 | 1,743.932 | 122.609 | -3.466 | -126.075 |
|  |  | [99.833] |  | [101.821] |  | [105.605] |  |  |  |
| **Migration background** | 132 | 0.152 | 118 | 0.246 | 121 | 0.182 | -0.094* | -0.030 | 0.064 |
|  |  | [0.031] |  | [0.040] |  | [0.035] |  |  |  |
| **Interpersonal Reactivity Index (IRI)** | 134 | 3.516 | 123 | 3.577 | 126 | 3.535 | -0.062 | -0.020 | 0.042 |
|  |  | [0.043] |  | [0.045] |  | [0.048] |  |  |  |
| **New Ecological Paradigm (NEP)** | 134 | 5.421 | 123 | 5.388 | 126 | 5.351 | 0.032 | 0.070 | 0.037 |
|  |  | [0.070] |  | [0.076] |  | [0.087] |  |  |  |
| **Racism** | 130 | 2.149 | 115 | 2.145 | 120 | 2.381 | 0.004 | -0.232 | -0.236 |
|  |  | [0.095] |  | [0.101] |  | [0.117] |  |  |  |
| **Political orientation** | 131 | 5.107 | 120 | 5.142 | 113 | 5.204 | -0.035 | -0.097 | -0.062 |
|  |  | [0.142] |  | [0.171] |  | [0.172] |  |  |  |
| **Flood experience** | 134 | 0.157 | 122 | 0.172 | 125 | 0.224 | -0.015 | -0.067 | -0.052 |
|  |  | [0.032] |  | [0.034] |  | [0.037] |  |  |  |
| **Education** | 134 | 5.918 | 123 | 5.878 | 126 | 5.905 | 0.040 | 0.013 | -0.027 |
|  |  | [0.108] |  | [0.115] |  | [0.123] |  |  |  |
| **Job** | 134 | 4.366 | 123 | 4.285 | 126 | 4.373 | 0.081 | -0.007 | -0.088 |
|  |  | [0.129] |  | [0.151] |  | [0.127] |  |  |  |
| **F-test of joint significance (F-stat)** | | |  |  |  |  | 0.294 | 0.786 | 0.750 |
| **F-test, number of observations** | | |  |  |  |  | 206 | 201 | 187 |
| *Note:* The value displayed for t-tests are the differences in the means across the groups. The value displayed for F-tests are the F-statistics. ***, **, and * indicate significance at the 1, 5, and 10 percent critical level. SE denotes standard errors (in brackets). | | | | | | | | | |
