# Supplementary material for: Distance to climate change consequences reduces willingness to engage in low-cost mitigation actions–Results from an experimental online study from Germany
Source: PLoS One. 2023 Apr 5;18(4):e0283190. doi: 10.1371/journal.pone.0283190 (PMC10075397; doi:10.1371/journal.pone.0283190)
Supplement: S2 Table — (DOCX) [file pone.0283190.s003.docx]

## S3 Table. Descriptive statistics.

| **VARIABLES/Conditions** | | **T Close Germany** | **T Far India** | **T Far Germany** | **(1)-(2)** | **(1)-(3)** | **(2)-(3)** |
| --- | --- | --- | --- | --- | --- | --- | --- |
| **Donation (yes)** | mean | 0.341 | 0.341 | 0.423 | 0.926 | 0.151 | 0.186 |
|  |  |  |  |  |  |  |  |
| **Donation (amount)** | mean | 0.992 | 0.992 | 1.309 | 0.494 | 0.554 | 0.087 |
|  | (sd) | (1.614) | (1.614) | (1.906) |  |  |  |
| **Petition (yes)** | mean | 0.167 | 0.167 | 0.276 | 0.006 | 0.516 | 0.037 |
|  |  |  |  |  |  |  |  |
| **Policy approval** | mean | 0.427 | 0.427 | 0.583 | 0.405 | 0.561 | 0.114 |
|  | (sd) | (0.791) | (0.791) | (0.710) |  |  |  |
| **N** |  | 126 | 126 | 123 |  |  |  |

*Note:* The non-parametric tests probing the equality in outcome variables between treatment conditions have been performed on basis of Chi2

tests for the dichotomous variables “Donation (yes)” and “Petition (yes)” and using Wilcoxon rank-sum tests for “Donation (amount)” and “Policy approval”.

The threshold of p ≤0.05 has been chosen in this paper as a cut-off for reported levels of statistical significance.
