# Supplementary material for: Distance to climate change consequences reduces willingness to engage in low-cost mitigation actions–Results from an experimental online study from Germany
Source: PLoS One. 2023 Apr 5;18(4):e0283190. doi: 10.1371/journal.pone.0283190 (PMC10075397; doi:10.1371/journal.pone.0283190)
Supplement: S3 Table — (DOCX) [file pone.0283190.s004.docx]

## S4 Table. Complementary analysis for Donations - Two-stage estimation.

|  | **(1)** | | **(2)** | |
| --- | --- | --- | --- | --- |
|  | **Probability** | **Amount** | **Probability** | **Amount** |
| **Far India** | -0.0695 | -0.0404 | -0.0782 | -0.0305 |
|  | (0.179) | (0.422) | (0.178) | (0.418) |
| **Far Germany** |  |  | 0.229 | 0.482 |
|  |  |  | (0.175) | (0.382) |
| **Disposable income (in EUR)** | -0.00005 | -0.0005* | 0.00001 | -0.0004* |
|  | (0.000) | (0.002) | (0.0001) | (0.0001) |
| **Flood experience** | 0.0345 | -0.679 | 0.0186 | -0.760 |
|  | (0.227) | (0.558) | (0.188) | (0.451) |
| **Migration background** | -0.137 | -0.717 | -0.172 | -1.064* |
|  | (0.246) | (0.627) | (0.192) | (0.479) |
| **Gender (not male)** | -0.0644 | -0.111 | 0.0424 | -0.344 |
|  | (0.182) | (0.427) | (0.148) | (0.331) |
| **Age** | 0.000520 | 0.0178 | 0.0002 | 0.0339* |
|  | (0.008 | (0.020) | (0.006) | (0.145) |
| **Constant** | -0.288 | 3.313*** | -0.417 | 2.747*** |
|  | (0.337) | (0.841) | (0.278) | (0.615) |
| **N** | 215 | | 315 | |
| Robust standard errors in parentheses, *** *p* < 0.01, ** *p* < 0.05, * *p* < 0.1. | | | | |

The table presents the regression coefficients from a linear Cragg–Hurdle model, which models the donation decision in two stages. At first, the probability of donation is estimated using a Probit regression model (with a lower hurdle of 0€). In the second stage, the donated amount is estimated with a Truncated-linear regression model, conditional on having decided to make the donation.
The two estimations correspond to Model (1) and Model (4) in Table 1 in the manuscript. Since this more elaborate analysis comes to the same conclusions, that both treatment conditions *Far Ger* and *Far India* do not have a significant impact on the decision to donate, nor on the amount to donate, we present the simpler analysis on basis of the binary donation decision in the main text.
