# Supplementary material for: Distance to climate change consequences reduces willingness to engage in low-cost mitigation actions–Results from an experimental online study from Germany
Source: PLoS One. 2023 Apr 5;18(4):e0283190. doi: 10.1371/journal.pone.0283190 (PMC10075397; doi:10.1371/journal.pone.0283190)
Supplement: S4 Table — (DOCX) [file pone.0283190.s005.docx]

## S5 Table. Regression results with sociodemographic controls and expected government support.

|  | **(1)** | **(2)** | **(3)** | **(4)** | **(5)** | **(6)** |
| --- | --- | --- | --- | --- | --- | --- |
|  | **Donation** | **Donation** | **Petition** | **Petition** | **Policy approval** | **Policy approval** |
| **Far India** | -0.0782 | -0.0026 | -0.4865* | -0.5084* | -0.0668 | -0.0109 |
|  | (0.178) | (0.209) | (0.196) | (0.224) | (0.105) | (0.123) |
| **Far Germany** | 0.2286 | 0.2286 | -0.018 | -0.0181 | 0.127 | 0.126 |
|  | (0.175) | (0.175) | (0.181) | (0.181) | (0.105) | (0.105) |
|  |  |  |  |  |  |  |
| **Disposable income (in EUR)** | 0.0000147 | 0.0000154 | -0.0000817 | -0.0000816 | -0.0001166 ** | -0.0001159** |
|  | (0.00007) | (0.00007) | (0.00007) | (0.00007) | (0.00004) | (0.00004) |
| **Flood experience** | 0.0186 | 0.0199 | 0.0375 | 0.0373 | -0.0486 | -0.0471 |
|  | (0.188) | (0.1883) | (0.020) | (0.020) | (0.112) | (0.112) |
| **Migration background** | -0.1723 | -0.1748 | 0.2823 | 0.2835 | 0.0440 | 0.0424 |
|  | (0.192) | (0.192) | (0.199) | (0.199) | (0.113) | (0.113) |
| **Gender (not male)** | 0.0424 | 0.0448 | 0.0618 | 0.0613 | 0.283** | 0.285** |
|  | (0.148) | (0.148) | (0.158) | (0.158) | (0.088) | (0.088) |
| **Age** | 0.0002 | 0.0002 | 0.0190** | 0.0190** | 0.0068 | 0.0068 |
|  | (0.006) | (0.006) | (0.006) | (0.006)) | (0.004) | (0.004) |
| **Expected government support** |  | 0.038 |  | -0.0115 |  | 0.0284 |
|  |  | (0.054) |  | (0.057) |  | (0.032) |
|  |  |  |  |  |  |  |
| **Constant** | -0.4171 | -0.6119 | -1.095*** | -1.037* | 0.337* | 0.193 |
|  | (0.278) | (0.394) | (0.296) | (0.414) | (0.165) | (0.232) |
| **N** | 315 | 315 | 315 | 315 | 315 | 315 |
| **p: Far Germany = Far India** | 0.093 | 0.277 | 0.020 | 0.031 | 0.076 | 0.280 |
| *Notes: This table shows the estimation results from regressing the impact of the treatment conditions Far India and Far Germany on the willingness to participate in mitigation actions, measured by three mitigation variables: Donation, Petition, and Policy approval. Model (1)–(2) and (4)–(5) show the coefficients probit regressions on the likelihood of a donation or petition. Models (5)-(6) are based on ordinary least squares regression models estimating the effect of the treatment conditions on the average approval of 12 realistic policy measures for climate protection in Germany. Model (2), (4) and (6) include as additional control the expected aid. Standard errors are indicated in parentheses. The symbols *, **, *** indicate significance at p<0.05, p<0.01, and p<0.001, respectively.* | | | | | | |

Model (1), (3) and (5) correspond with Model (4)-(6) of Table 1 in the main text. Model (2), (4) and (6) control in addition for participants’ expectations about whether the flooding victims will receive support through the government. As one can see, adding government support as a further control did not impact our findings. There remains a significant negative effect of T_Far India_ on the willingness to sign the petition.
