# Supplementary material for: Distance to climate change consequences reduces willingness to engage in low-cost mitigation actions–Results from an experimental online study from Germany
Source: PLoS One. 2023 Apr 5;18(4):e0283190. doi: 10.1371/journal.pone.0283190 (PMC10075397; doi:10.1371/journal.pone.0283190)
Supplement: S5 Table — (DOCX) [file pone.0283190.s006.docx]

## S6 Table. Regression results for perceived personal affectedness.

|  | | | **Perceived personal affectedness** | | |
| --- | --- | --- | --- | --- | --- |
|  | **(1)** | **(2)** | | **(3)** | **(4)** |
|  | **Affectedness** | **Petition** | | **Petition** | **Petition** |
|  |  |  | |  |  |
| **Far India** | -0.527** | -0.487* | |  | -0.4419* |
|  | (0.196) | (0.196) | |  | (0.198) |
|  |  |  | |  |  |
| **Far Germany** | 0.370 | -0.018 | |  | -0.0557 |
|  | (0.196) | (0.181) | |  | (0.183) |
|  |  |  | |  |  |
| **Affectedness** |  |  | | 0.117* | 0.0920 |
|  |  |  | | (0.054) | (0.0563) |
|  |  |  | |  |  |
| Constant | 3.663*** | -1.095*** | | -1.670*** | -1.434*** |
|  | (0.307) | (0.296) | | (0.347) | (0.364) |
| N | 315 | 315 | | 315 | 315 |
| Controls included | yes | yes | | yes | yes |
| p: Far India=Far Germ | <0.001 | 0.020 | |  | 0.064 |
| *Notes: Regression models presented in this table examine whether an altered feeling of personal affectedness mediated the treatment effect on the willingness to sign the petition. Model (1) is based on ordinary least square regression models, examining whether affectedness was influenced by the treatments. Model (2)-(4) estimate the likelihood of signing the petition, employing a probit model. Model (2) is the same as Model (5) of Table 1 in the main text. Model (3) assesses the impact of perceived personal affectedness on the willingness to sign the petition. Model (4) then includes perceived personal affectedness in the original estimation of Model (2) and assesses whether affectedness mediated the effect of the treatment conditions. Controls included are sociodemographic characteristics such as age, gender, disposable income, migration background, as well as own flood experience. Standard errors are indicated in parentheses. The symbols *, **, *** indicate significance for p<0.05, p<0.01, and p<0.001, respectively.* | | | | | |
