# Supplementary material for: Distance to climate change consequences reduces willingness to engage in low-cost mitigation actions–Results from an experimental online study from Germany
Source: PLoS One. 2023 Apr 5;18(4):e0283190. doi: 10.1371/journal.pone.0283190 (PMC10075397; doi:10.1371/journal.pone.0283190)
Supplement: S6 Table — (DOCX) [file pone.0283190.s007.docx]

## S7 Table. Regression results with interaction terms for high racism.

|  | **(1)** | **(2)** | **(3)** |
| --- | --- | --- | --- |
|  | **Donation** | **Petition** | **Policy approval** |
| **Far India** | -0.0011 | -0.4315* | -0.0069 |
|  | (0.188) | (0.206) | (0.109) |
|  |  |  |  |
| **Far Germany** | 0.2859 | -0.0251 | 0.2081 |
|  | (0.183) | (0.189) | (0.108) |
|  |  |  |  |
| **High racism** | -0.5394 | -0.290 | -0.1498 |
|  | (0.473) | (0.487 | (0.250) |
| **Far India # High racism** | -0.6064 | -0.3868 | -0.3415 |
|  | (0.709) | (0.723) | (0.330) |
| **Far Germany # High racism** | -0.6759 | 0.1619 | -0.7682* |
|  | (0.733) | (0.656 | (0.354) |
|  |  |  |  |
| **Disposable income (in EUR)** | .0000196 | -.0000753 | -.0001184** |
|  | (0.00007) | (0.00007) | (0.00004) |
| **Flood experience** | 0.0855 | 0.0645 | -0.0146 |
|  | (0.193) | (0.202) | (0.110) |
| **Migration background** | -0.1866 | 0.2780 | 0.0537 |
|  | (0.195) | (0.200) | (0.111) |
| **Gender (not male)** | -0.0124 | 0.0441 | 0.239** |
|  | (0.151) | (0.160) | (0.087) |
| **Age** | 0.0013 | 0.020 | 0.008* |
|  | (0.006) | (0.007) | (0.004) |
|  |  |  |  |
| **Constant** | -0.411 | -1.107*** | 0.320 |
|  | (0.288) | (0.302) | (0.163) |
| **N** | 315 | 315 | 315 |
| **p: Far Germany = Far India** | 0.136 | 0.056 | 0.058 |
| **p: Far Germany # High racism = Far India # High racism** | 0.928 | 0.429 | 0.197 |
| *Notes: This table shows the estimation results from regressing the treatment conditions Far India and Far Germany on the willingness to participate in mitigation actions, under consideration of an interaction with the dummy variable High racism, taking the value one when the individual's score was above the 90th percentile on the racism index. The three mitigation variables were donation (Model(1)), petition (Model(2)), and policy approval (Model(3)). Standard errors are indicated in parentheses. The symbols *, **, *** indicate significance at p<0.05, p<0.01, and p<0.001, respectively.* | | | |
