# Supplementary material for: Distance to climate change consequences reduces willingness to engage in low-cost mitigation actions–Results from an experimental online study from Germany
Source: PLoS One. 2023 Apr 5;18(4):e0283190. doi: 10.1371/journal.pone.0283190 (PMC10075397; doi:10.1371/journal.pone.0283190)
Supplement: S7 Table — (DOCX) [file pone.0283190.s008.docx]

## S8 Table. Racism as predictor for mitigation actions.

|  | **(1)** | **(2)** | **(3)** |  |
| --- | --- | --- | --- | --- |
|  | **Donation** | **Petition** | **Policy approval** |  |
| **Racism** | -0.2605*** | -0.183* | -0.211*** |  |
|  | (0.072) | (0.075) | (0.037) |  |
|  |  |  |  |  |
| **Disposable income (in EUR)** | 0.0000149 | -0.000122 | -0.0001334 *** |  |
|  | (-0.00007) | (-0.00007) | (-0.00004) |  |
| **Flood experience** | 0.1265 | 0.0405 | -0.0053 |  |
|  | (0.1956) | (0.207) | (0.109) |  |
| **Migration background** | -0.2028 | 0.2365 | 0.0352 |  |
|  | (0.197) | (0.202) | (0.108) |  |
| **Gender (not male)** | -0.0460 | -0.0415 | 0.1929* |  |
|  | (0.152) | (0.162) | (0.0848) |  |
| **Age** | 0.0026 | 0.0234*** | 0.0087* |  |
|  | (0.006)) | (0.007) | (0.004) |  |
|  |  |  |  |  |
| **Constant** | 0.154 | -0.877** | 0.833*** |  |
|  | (0.306) | (0.318)) | (0.167) |  |
| **N** | 306 | 306 | 306 |  |
| *Notes: This table shows the estimation results from regressing the impact of racism on the willingness to participate in mitigation actions, measured by three mitigation variables: donation (Model(1)), petition (Model(2)), and policy approval (Model(3)). Standard errors are indicated in parentheses. The symbols *, **, *** indicate significance at p<0.05, p<0.01, and p<0.001, respectively.* | | | | |
